# Radiosensitization of noradrenaline transporter-expressing tumour cells by proteasome inhibitors and the role of reactive oxygen species

**DOI:** 10.1186/2191-219X-3-73

**Published:** 2013-11-13

**Authors:** Colin Rae, Mathias Tesson, John W Babich, Marie Boyd, Robert J Mairs

**Affiliations:** 1 Radiation Oncology, Institute of Cancer Sciences, University of Glasgow, Garscube Estate, Glasgow G61 1BD, Scotland; 2 Department of Radiology, Cornell University, Ithaca, NY 14850, USA; 3 I1nstitute of Pharmacy and Biomedical Sciences, Strathclyde University, Glasgow G4 0RE, UK

**Keywords:** Bortezomib, Proteasome, ^131^I-metaiodobenzylguanidine, Neuroblastoma, Radiosensitizer

## Abstract

**Background:**

The radiopharmaceutical ^131^I-metaiodobenzylguanidine (^131^I-MIBG) is used for the targeted radiotherapy of noradrenaline transporter (NAT)-expressing neuroblastoma. Enhancement of ^131^I-MIBG's efficacy is achieved by combination with the topoisomerase I inhibitor topotecan - currently being evaluated clinically. Proteasome activity affords resistance of tumour cells to radiation and topoisomerase inhibitors. Therefore, the proteasome inhibitor bortezomib was evaluated with respect to its cytotoxic potency as a single agent and in combination with ^131^I-MIBG and topotecan. Since elevated levels of reactive oxygen species (ROS) are induced by bortezomib, the role of ROS in tumour cell kill was determined following treatment with bortezomib or the alternative proteasome inhibitor, MG132.

**Methods:**

Clonogenic assay and growth of tumour xenografts were used to investigate the effects of proteasome inhibitors alone or in combination with radiation treatment. Synergistic interactions *in vitro* were evaluated by combination index analysis. The dependency of proteasome inhibitor-induced clonogenic kill on ROS generation was assessed using antioxidants.

**Results:**

Bortezomib, in the dose range 1 to 30 nM, decreased clonogenic survival of both SK-N-BE(2c) and UVW/NAT cells, and this was prevented by antioxidants. It also acted as a sensitizer *in vitro* when administered with X-radiation, with ^131^I-MIBG, or with ^131^I-MIBG and topotecan. Moreover, bortezomib enhanced the delay of the growth of human tumour xenografts in athymic mice when administered in combination with ^131^I-MIBG and topotecan. MG132 and bortezomib had similar radiosensitizing potency, but only bortezomib-induced cytotoxicity was ROS-dependent.

**Conclusions:**

Proteasome inhibition shows promise for the treatment of neuroblastoma in combination with ^131^I-MIBG and topotecan. Since the cytotoxicity of MG132, unlike that of bortezomib, was not ROS-dependent, the latter proteasome inhibitor may have a favourable toxicity profile in normal tissues.

## Background

Neuroblastoma, the most common solid extra-cranial tumour in children, accounts for approximately 15% of all childhood cancer deaths. It is a disease of the postganglionic sympathetic nervous system which commonly arises in the adrenal gland. Most neuroblastoma cells express the noradrenaline transporter (NAT), a characteristic that enables diagnostic imaging and therapy using the radiolabelled noradrenaline analogue metaiodobenzylguanidine - ^123^I-MIBG and ^131^I-MIBG, respectively. Approximately 95% of tumours show affinity for MIBG [1]. Although ^131^I-MIBG is associated with therapeutic success in the form of long-term remissions and palliation, it is likely that for maximum efficacy, there is a requirement for this radiopharmaceutical to be administered in combination with other chemotherapeutic agents [2]. We have previously demonstrated the potential use of ^131^I-MIBG in combination with the topoisomerase I inhibitor topotecan [3,4], the poly(ADP-ribose) polymerase (PARP) inhibitor PJ34 [5] and disulfiram [6].

There is growing interest in targeting the proteasome for anti-cancer therapy. Abnormally high proteasome expression and activity are observed in many cancer cells and are closely related to cellular proliferation [7]. Proteolysis by the 26S proteasome is an essential metabolic process which regulates the degradation of tumour suppressors, transcription factors and proteins involved in cell cycle control as well as mutant and damaged proteins. Inhibition of proteasome function causes the abnormal accumulation of many intracellular proteins, resulting in cell cycle arrest and apoptosis [8].

Cancer cells are more sensitive to the modulation of proteasome activity than normal cells and proteasome inhibition increases the sensitivity of cancer cells to various anti-cancer agents [9]. As well as having efficacy as single agents, proteasome inhibitors have been demonstrated to enhance the anti-tumour activity of other drugs, including inhibitors of topoisomerase I [10] and histone deacetylase [11]. Proteasome inhibition also sensitizes cancer cells to radiation by down-regulation of the DNA damage response [12], by prevention of the activation of radiation-induced nuclear factor-κB (NF-κB) [9] and through cell cycle arrest in the radiosensitive G2/M phase [13].

Bortezomib is the first proteasome inhibitor approved by US FDA for the treatment of multiple myeloma. This drug has been demonstrated to suppress tumour growth and angiogenesis in solid tumours including breast, prostate, lung, neuroblastoma, and mesothelioma [14]. Bortezomib's sensitization of cancer cells to radiation treatment [15,16] also suggests it may be suitable for combination with ^131^I-MIBG therapy in neuroblastoma patients. Indeed, bortezomib has recently been used in combination with ^90^Y-ibritumomab or ^153^Sm-lexidronam for the treatment of non-Hodgkin lymphoma or multiple myeloma, respectively [17,18]. In experimental models of neuroblastoma, bortezomib has been shown to inhibit cell proliferation; increase survival of human tumour xenografts in athymic mice; inhibit angiogenesis [19,20]; and enhance the cytotoxicity of topotecan [10], docetaxel [21], and retinoids [22]. Acquired drug resistance is an important cause of neuroblastoma treatment failure and relapse [23]. Encouragingly, bortezomib is not a substrate for multidrug resistance-associated proteins [19], and it induces cell death regardless of p53 status [20]. Furthermore, in children, bortezomib is associated with minimal systemic toxicity [24].

Normal cells have relatively low concentrations of reactive oxygen species (ROS) and high antioxidant capacity, whereas cancer cells generate abnormally high levels of ROS due to aberrant metabolism [25]. Bortezomib-induced apoptotic signalling in cultured human cancer cells is initiated by ROS, and apoptosis is prevented by administration of antioxidants [26]. Bortezomib-induced ROS generation may also be responsible for some side effects associated with the drug which currently limit its clinical use. In particular, peripheral neuropathy, which can affect up to 30% of patients receiving chemotherapy, may be induced by ROS [27]. Therefore, in order to minimise normal tissue toxicity, it is necessary to characterize the mode of action of bortezomib and alternative proteasome inhibitors.

In this study, we determined the capacity of bortezomib to enhance the sensitivity of NAT-expressing cells to radiotherapy in the form X-radiation or ^131^I-MIBG. Furthermore, triple combination therapy, consisting of bortezomib with ^131^I-MIBG + topotecan, was evaluated in comparative investigations. We also compared the mechanisms of cytotoxicity of bortezomib with a different class of proteasome inhibitor, MG132, with respect to dependence on ROS-induced cell death.

## Methods

### Reagents

Bortezomib was a gift from Millenium Pharmaceuticals (Cambridge, MA, USA), MG132 was purchased from Sigma-Aldrich (Dorset, UK) and topotecan from Axxora UK Ltd. (Nottingham, UK). All cell culture media and supplements were purchased from Life Technologies Ltd. (Paisley, UK), and all other chemicals were from Sigma-Aldrich (Dorset, UK). No-carrier-added ^131^I-MIBG was prepared using a solid-phase system wherein the precursor of MIBG was attached to an insoluble polymer via the tin-aryl bond [28].

### Cell culture

Human neuroblastoma-derived SK-N-BE(2c) cells were purchased from the American Type Culture Collection (Manassas, VA, USA). The UVW cell line was derived from a human glioblastoma [29]. Cell lines were authenticated in-house using the AmpF/STR Identifiler kit (Applied Biosytems, Warrington, UK). SK-N-BE(2c) cells were maintained in DMEM containing 15% (*v*/*v*) fetal calf serum (FCS). UVW cells were transfected to express the NAT gene, facilitating the active uptake of MIBG, as previously described [30] and were maintained in MEM, containing 10% (*v*/*v*) FCS and 1 mg/ml geneticin. Transfectants were designated as UWV/NAT.

### Clonogenic survival assay

Cells were seeded in 25-cm^2^ flasks at 10^5^ cells/flask. When cultures were in exponential growth phase, medium was removed and replaced with fresh medium containing the proteasome inhibitors bortezomib or MG132, the antioxidants *N*-acetyl-*L*-cysteine (NAC, 1 mM) or tiron (4,5-Dihydroxy-1,3-benzenedisulfonic acid disodium salt monohydrate, 1 mM), or various combinations of these agents. This enabled a comparison of NAC and tiron with respect to the contribution of ROS generation to the cytotoxicity of the proteasome inhibitors. Cells were incubated with drugs for 24 h at 37 C in 5% CO_2_. In separate treatments, cells were exposed to X-rays using an RS225 irradiator (Xstrahl, Surrey, UK) at a dose-rate of 1.33 Gy/min, then incubated for 24 h at 37°C in 5% CO_2_. After treatment, cells were seeded for clonogenic assay as previously described [3,4].

### Combination treatments

The cytotoxic interaction between bortezomib and radiation was examined using clonogenic assay and combination index analysis, according to the method of Chou and Talalay [31]. In this analysis, the toxicity induced by single drugs and scheduled combinations is investigated using the equation CI = (*D*)_1_/(*Dx*)_1_ + (*D*)_2_/(*Dx*)_2_, where (*D*)_1_ and (*D*)_2_ are the doses of each agent which inhibit *x*% of cell growth when used in combination and (*Dx*)_1_ and (*Dx*)_2_ are the doses of each drug which inhibit *x*% of colonies when used as single agents.

Initially, exponentially growing cells were treated with each agent alone to determine effective doses. Cells were subsequently treated with a range of doses of bortezomib and radiation, using a fixed dose ratio of bortezomib to radiation, so that the proportional contribution of each agent in the mixtures would be the same at all treatment intensities. The fixed dose ratio was equivalent to 7.6 nM bortezomib:3.8 Gy X-radiation, based on their respective IC_50_ values. For combinations of ^131^I-MIBG and bortezomib, the fixed dose ratio was 7.6 nM bortezomib:1.5 MBq ^131^I-MIBG. For the purposes of combination index analysis, simultaneous treatment with {^131^I-MIBG + topotecan} was considered as one agent, and the fixed dose ratio was 7.6 nM:0.5 MBq:4.9 nM (bortezomib:^131^I-MIBG:topotecan), as this dose killed 50% of clonogens when administered simultaneously in combination. Three different treatment schedules were assessed: bortezomib given 24 h before, after or simultaneously with radiation. The effectiveness of combinations of bortezomib and radiation was quantified by determining a combination index (CI) at various levels of cytotoxicity. CI < 1, CI = 1 and CI >1 indicate synergism, additivity and antagonism, respectively.

### Tumour xenografts

Six-week-old female, congenitally athymic mice of strain CD1 *nu/nu* were obtained from Charles River plc (Kent, UK). *In vivo* experiments were carried out in accordance with the Animals (Scientific Procedures) Act 1986. Tumours in athymic mice formed from SK-N-BE(2c) and UVW/NAT cells express the NAT enabling active uptake of ^131^I-MIBG. Subcutaneous tumour growth was established as previously described [3]. Mice were used for experimental therapy when the tumour volumes had reached approximately 100 mm^3^. To monitor potential toxicity, experimental animals were examined daily for signs of distress and weighed weekly. Mice were randomized into treatment groups, each consisting of six animals that received: PBS solution; 0.8 mg/kg bortezomib solution; simultaneous administration of ^131^I-MIBG (18 or 5 MBq for SK-N-BE(2c) or UVW/NAT, respectively) and topotecan (1.75 or 0.875 mg/kg for SK-N-BE(2c) or UVW/NAT, respectively); or administration of bortezomib 24 h after {^131^I-MIBG + topotecan} - all by i.p injection. The indicated activities of ^131^I-MIBG administered to the mice were shown previously by us to induce significant delay of growth but incomplete sterilization of SK-N-BE(2c) and UVW/NAT xenografts, and the simultaneous administration of ^131^I-MIBG + topotecan was demonstrated to be the most effective schedule [3]. Bortezomib doses (0.5 to 1 mg/kg) were similar to those used in previous preclinical studies [9]. Tumours were measured with callipers immediately before treatment and twice weekly thereafter. On the assumption of ellipsoidal geometry, diameter measurements were converted to an approximate tumour volume by multiplying half the longest diameter by the square of the mean of the two shorter diameters.

### Statistics

Data are presented as means ± standard error of the mean (SEM), unless otherwise stated, with the number of independent repetitions provided in the legend to each figure. Statistical significance was determined using Student's *t* test. A *P* value < 0.05 was considered to be statistically significant and < 0.01 highly significant.

## Results

### Bortezomib is a radiosensitizer

**Figure 1:**
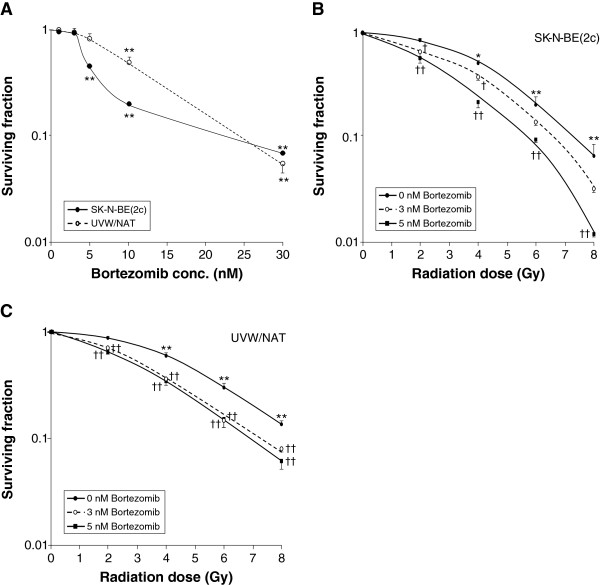
**Cytotoxicity and radiosensitizing effect of bortezomib. (A)** Clonogenic survival after exposure of SK-N-BE(2c) cells or UVW/NAT cells to bortezomib for 24 h. Clonogenic survival of **(B)** SK-N-BE(2c) cells and **(C)** UVW/NAT cells simultaneously exposed to X-radiation and 3 or 5 nM bortezomib for 24 h. Data are means ± SEM, *n* = 4; significance of differences: * *p* < 0.05, ** *p* < 0.01 from untreated control, † *p* < 0.05, †† *p* < 0.01 from radiation treatment alone.

When given as a single agent at a dose of 1 to 30 nM, bortezomib decreased the survival of clonogens of both SK-N-BE(2c) cells and UVW/NAT cells (Figure 1A) in a concentration-dependent manner. Following treatment of either cell line with bortezomib at concentrations ≥ 10 nM, clonogenic cell kill was highly significantly greater than that of untreated control cultures. The decreased clonogenic capacity of SK-N-BE(2c) and UVW/NAT cells resulting from X-irradiation was enhanced by the treatment with bortezomib at 3 and 5 nM (Figures 1B,C). The IC_50_ values obtained for SK-N-BE(2c) cells exposed to X-radiation alone, or in the presence of 3 or 5 nM bortezomib were 3.72 ± 0.16, 2.82 ± 0.20 and 2.42 ± 0.15 Gy, respectively. For UVW/NAT cells, the IC_50_ values were 4.23 ± 0.02, 2.94 ± 0.12 and 2.73 ± 0.11 Gy for X-radiation alone and 3 and 5 nM bortezomib, respectively. These results indicate dose enhancement ratios at the 50% level of toxicity (DER_50_), in SK-N-BE(2c) cells and UVW/NAT cells respectively, of 1.44 and 1.32 for 3 nM bortezomib, and 1.54 and 1.55 for 5 nM bortezomib.

Representative CI values for treatment of SK-N-BE(2c) and UVW/NAT cells with bortezomib and X-radiation are shown in Table 1. These indicated that supra-additive clonogenic cell kill (CI < 1) was dependent on both the dose and the schedule. Synergism was most appreciable following treatment with high dosage of both agents and the administration of bortezomib 24 h after X-radiation (Table 1).

**Table 1:** Synergism analysis of various schedules of administration of bortezomib and X-radiation

**Effect level**	Combination index*			Combination index*		
	**SK-N-BE(2c) cells**			**UVW/NAT cells**		
	**Simultaneous**	**BZ before**	**BZ after**	**Simultaneous**	**BZ before**	**BZ after**
ED_25_	1.12	1.16	1.16	1.02	*0.90*	*0.77*
ED_50_	*0.94*	1.04	*0.95*	*0.95*	*0.84*	*0.79*
ED_75_	*0.80*	*0.93*	*0.78*	*0.89*	*0.80*	*0.80*

Asterisk '*’ indicates that the combination index values are means of four experiments. They were derived from median effect analysis, according to independent action model, for three different levels of toxicity and three alternative schedules of administration. CI values < 1 indicate synergy and values > 1 indicate partial antagonism. Administration schedules were simultaneous, bortezomib 24 h before X-radiation (BZ before) and bortezomib 24 h after X-radiation (BZ after). Italicised values indicate synergy.

Similarly, the interaction between bortezomib and ^131^I-MIBG suggested that the administration schedule was an important determinant of synergism. For SK-N-BE(2c) and UVW/NAT cells, synergism was evident in response to the administration of bortezomib 24 h after ^131^I-MIBG or simultaneous treatment with both agents (Table 2). Administration of bortezomib 24 h before ^131^I-MIBG had an antagonistic effect (CI > 1) upon the toxicity to both cell lines at all combination doses.

**Table 2:** **Synergism analysis of various schedules of administration of bortezomib and **^**131**^**I-MIBG**

**Effect level**	Combination index*			Combination index*		
	**SK-N-BE(2c) cells**			**UVW/NAT cells**		
	**Simultaneous**	**BZ before**	**BZ after**	**Simultaneous**	**BZ before**	**BZ after**
ED_25_	*0.82*	1.34	*0.73*	*0.56*	1.14	*0.40*
ED_50_	*0.65*	1.30	*0.63*	*0.70*	1.11	*0.49*
ED_75_	*0.94*	2.26	*0.96*	1.48	1.86	1.08

Asterisk '*’ indicates that the combination index values are means of four experiments. Administration schedules were simultaneous, bortezomib 24 h before ^131^I-MIBG (BZ before) and bortezomib 24 h after ^131^I-MIBG (BZ after). Italicised values indicate synergy.

Three-way combination treatment consisted of bortezomib and {^131^I-MIBG + topotecan} - the latter two agents being given simultaneously [3]. Supra-additive clonogenic cell kill was observed only when bortezomib was administered 24 h after {^131^I-MIBG + topotecan} (Table 3).

**Table 3:** **Synergism analysis of various schedules of administration of bortezomib {**^**131**^**I-MIBG + topotecan}**

**Effect level**	Combination index*			Combination index*		
	**SK-N-BE(2c) cells**			**UVW/NAT cells**		
	**Simultaneous**	**BZ before**	**BZ after**	**Simultaneous**	**BZ before**	**BZ after**
ED_25_	1.46	1.42	*0.64*	1.30	1.71	*0.77*
ED_50_	1.54	1.70	*0.63*	1.44	2.02	*0.77*
ED_75_	1.63	2.04	*0.63*	1.60	2.41	*0.79*

Asterisk '*’ indicates that the combination index values are means of four experiments. Administration schedules were simultaneous, bortezomib 24 h before {^131^I-MIBG + topotecan} (BZ before) and bortezomib 24 h after {^131^I-MIBG + topotecan} (BZ after). Italicised values indicate synergy.

### Bortezomib enhances tumour growth delay

**Figure 2:**
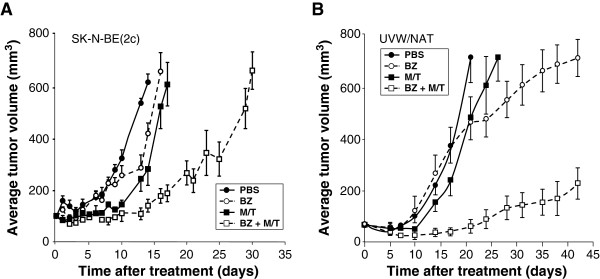
**Effect of bortezomib alone or combined with **^**131**^**I-MIBG/topotecan on the growth delay of experimental tumours.** Growth of human tumour xenografts derived from **(A)** SK-N-BE(2c) cells or **(B)** UVW/NAT cells in athymic mice exposed to PBS, bortezomib alone (0.8 mg/kg), ^131^I-MIBG (18 or 5 MBq for SK-N-BE(2c) or UVW/NAT, respectively) + topotecan (1.75 or 0.875 mg/kg for SK-N-BE(2c) or UVW/NAT, respectively) or the combination of bortezomib with {^131^I-MIBG + topotecan}. Data are expressed as mean tumour volume at every time point (mm^3^) ± SD. Abbreviations: BZ, bortezomib; M/T, ^131^I-MIBG and topotecan administered simultaneously.

*In vitro* experimental results indicated no enhancement of tumour cell kill by scheduling bortezomib 24 h before {^131^I- MIBG + topotecan} or simultaneous administration of the components of the triple combination. Therefore, in order to reduce the number of experimental animals, the latter schedules were not administered to athymic mice bearing xenografts. None of the animals in this study showed sign of distress. The effects of agents administered alone or in combination on the growth in athymic mice of xenografts derived from SK-N-BE(2c) and UVW/NAT cells are shown in Figure 2. Bortezomib alone induced a slight delay in the growth of SK-N-BE(2c) tumours. The time taken to increase tumour volume fivefold (*τ*_5_) was 14.7 days, compared to untreated control *τ*_5_ of 12.3 days. Simultaneous administration of {^131^I-MIBG + topotecan} induced a similar delay in the growth of SK-N-BE(2c) xenografts (*τ*_5_ = 15.8 days). However, bortezomib administered 24 h after {^131^I-MIBG + topotecan} resulted in enhanced tumour growth delay, manifest by a *τ*_5_ value of 28.5 days.

In xenografts derived from UVW/NAT cells, bortezomib alone had no effect on growth rate, exemplified by *τ*_5_ values of 16.0 and 15.9 days for untreated control groups and bortezomib, respectively. In contrast, simultaneous administration of ^131^I-MIBG and topotecan induced an enhancement of growth delay (*τ*_5_ = 18.2 days) compared with PBS-treated controls. Bortezomib administered 24 h after {^131^I- MIBG + topotecan} resulted in a failure by tumours to achieve a fivefold increase in volume over 42 days. Therefore, in xenografts derived from either SK-N-BE(2c) or UVW/NAT cells, the triple combination, consisting of bortezomib administered 24 h after {^131^I- MIBG + topotecan}, induced significantly greater growth delay than bortezomib alone or the ^131^I-MIBG + topotecan combination.

### Bortezomib-induced clonogenic kill is ROS-dependent

**Figure 3:**
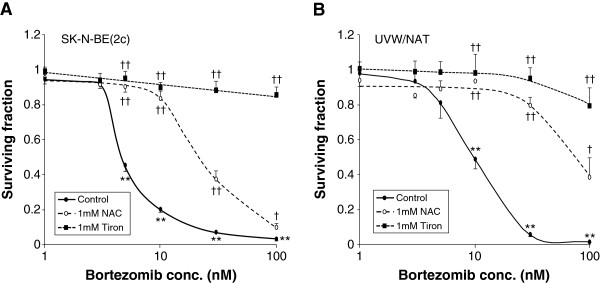
**Effect of antioxidants on bortezomib-induced clonogenic cell kill.** Clonogenic assay of **(A)** SK-N-BE(2c) and **(B)** UVW/NAT cells exposed to bortezomib for 24 h in the presence or absence of the antioxidants NAC (1 mM) or tiron (1 mM). Data are means ± SEM, *n* = 4; significance of differences: ** *p* < 0.01 from untreated control, † *p* < 0.05, †† *p* < 0.01 from bortezomib treatment alone.

The mechanism of bortezomib-induced clonogenic cell kill was investigated by determining the protection afforded by treatment with antioxidants. The results are shown in Figure 3. In both SK-N-BE(2c) and UVW/NAT cells, the magnitude of bortezomib-induced clonogenic cell kill was diminished by NAC. For example, exposure to 10 nM bortezomib reduced clonogenic survival to 20% (SK-N-BE(2c) cells) or 49% (UVW/NAT cells) of control values, whereas in the presence of 1 mM NAC, the corresponding values were 83% and 93%, respectively. This suggests that a significant proportion of the cytotoxicity induced by bortezomib as a single agent was due to ROS. An alternative ROS scavenger, tiron, completely blocked bortezomib-induced cytotoxicity at all concentrations (Figure 3), suggesting a different mechanism of action of NAC and tiron or a different degree of nullification of ROS.

### Radiosensitization by MG132 is not ROS-dependent

**Figure 4:**
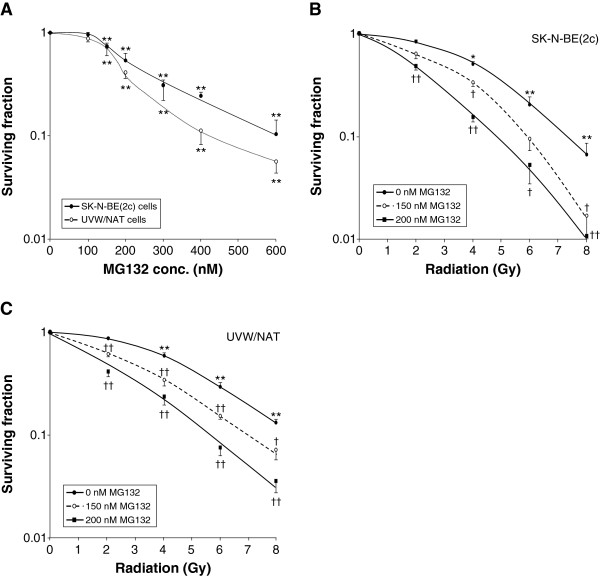
**Cytotoxicity and radiosensitizing effect of MG132. (A)** Clonogenic assay of SK-N-BE(2c) and UVW/NAT cells exposed to MG132 for 24 h. Clonogenic assay of **(B)** SK-N-BE(2c) and **(C)** UVW/NAT cells exposed to X-radiation and 150 nM or 200 nM MG132 for 24 h. Data are means ± SEM, *n* = 4; significance of differences: * *p* < 0.05, ** *p* < 0.01 from untreated control, † *p* < 0.05, †† *p* < 0.01 from radiation treatment alone.

MG132, an alternative inhibitor of proteasome activity, may have a pharmacologic profile different from that of bortezomib. Single agent treatment with MG132 caused a concentration-dependent reduction in the survival of SK-N-BE(2c) and UVW/NAT clonogens (Figure 4A). MG132 also sensitized both cell lines to radiation treatment (Figure 4B,C). The IC_50_ values obtained for SK-N-BE(2c) cells following X-irradiation alone or with simultaneous administration of 150 or 200 nM MG132 were 3.72 ± 0.17, 2.70 ± 0.18 and 2.15 ± 0.09 Gy, respectively. For irradiated UVW/NAT cells, the corresponding IC_50_ values were 4.23 ± 0.16, 2.36 ± 0.09 and 2.00 ± 0.20 Gy. These results indicate dose enhancement ratios at the 50% level of cell kill (DER_50_), in SK-N-BE(2c) cells and UVW/NAT cells, respectively, of 1.38 and 1.79 for 150 nM MG132, and 1.73 and 2.12 for 200 nM MG132. These DER_50_ values are comparable to those obtained following treatment with bortezomib, which ranged from 1.32 to 1.55. This suggests that although the two agents had a similar effect, the concentrations required differed by approximately 40-fold. This difference in potency between bortezomib and MG132 has previously been reported [13,32,33], though not fully explained.

**Figure 5:**
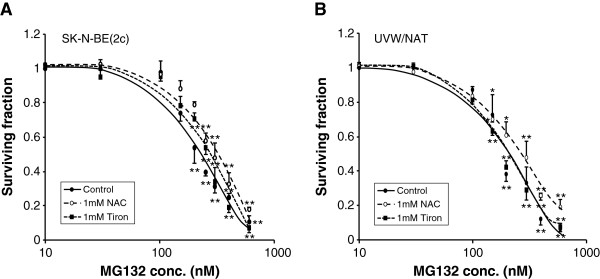
**Effect of antioxidants on MG132-induced clonogenic cell kill.** Clonogenic assay of **(A)** SK-N-BE(2c) and **(B)** UVW/NAT cells exposed to MG132 for 24 h in the presence or absence of NAC (1 mM) or tiron (1 mM). Data are means ± SEM, *n* = 4; significance of differences: * *p* < 0.05, ** *p* < 0.01 from untreated control.

The dose-dependent kill of SK-N-BE(2c) or UVW/NAT clonogens observed following treatment with MG132 was not significantly altered by simultaneous treatment with the antioxidants NAC or tiron (Figure 5). Therefore, in contrast to the clonogenic cell kill resulting from bortezomib treatment, MG132-induced kill was not mediated by ROS. This suggests that although bortezomib and MG132 both target the proteasome, both induce clonogenic kill as single agents and both sensitize cancer cells to ionizing radiation, their mechanisms of cytotoxicity do not both involve the generation of ROS.

## Discussion

In agreement with previous reports of the cytotoxicity of the proteasome inhibitors bortezomib and MG132 *in vitro*[19,34], we observed that treatment with these drugs, as single agents, induced concentration-dependent decreases in the survival of clonogens from two tumour cell lines - SK-N-BE(2c) and UVW/NAT. Proteasome inhibition may cause growth arrest and cell death by several mechanisms of action including inhibition of the expression of NF-κB-dependent, anti-apoptotic genes [32] and accumulation of pro-apoptotic proteins [21]. Bortezomib may also overcome multidrug resistance in relapsed neuroblastoma [20].

Proteasome inhibitors have been reported to be radiosensitzers *in vitro*[15,16,35,36]. This therapeutic activity was supported by our observation of enhanced radiation cell kill in the presence of bortezomib or MG132. The combination of proteasome inhibitor with X-radiation engendered synergistic enhancement of clonogenic kill, as assessed by combination index analysis (CI values of less than 1) and enhancement of radiation kill (dose enhancement ratios between 1.54 and 2.12).

It has been observed, using *in vitro* and *in vivo* models, that bortezomib enhanced topotecan experimental therapy in neuroblastoma [10]. Furthermore, we have previously demonstrated synergistic interaction between ^131^I-MIBG and topotecan [3,4]. The present results indicate not only that bortezomib improved ^131^I-MIBG therapy but also that a triple combination comprising bortezomib, ^131^I-MIBG and topotecan enhanced clonogenic cell kill *in vitro* and delayed the growth of NAT-expressing tumour xenografts *in vivo*. This was possible both *in vitro* and *in vivo* using concentrations of bortezomib which were clinically achievable [37]. These studies also demonstrated the importance of drug scheduling. According to combination index analysis of clonogenic survival *in vitro*, the administration of bortezomib prior to {^131^I-MIBG and topotecan} produced no supra-additive cytotoxicity (CI value greater than 1) whereas treatment with {^131^I-MIBG and topotecan} 24 h before bortezomib proved to be synergistic in the treatment of both SK-N-BE(2c) and UVW/NAT cells. The efficacy of the latter schedule was confirmed by the enhanced delay of the growth of xenografts derived from SK-N-BE(2c) or UVW/NAT cells compared with that achieved by bortezomib alone or by the double combination of ^131^I-MIBG and topotecan. The absence of synergism following the administration of bortezomib before radiation suggests that DNA damage and/or NF-κB activation is necessary before the benefit of proteasome inhibition becomes apparent.

In response to ionizing radiation, NF-κB activation is elevated and this is likely to be prevented by proteasome inhibition [32]. Moreover, proteasome inhibitors have radiosensitizing activity which occurs independently of NF-κB activity, via disruption of the balance between pro- and anti-apoptotic signalling [21], loss of DNA repair [12] and inhibition of cell cycle progression [13]. The accumulation of reactive oxygen species (ROS) resulting from exposure to ionizing radiation may also be increased by NF-κB inhibition, further supporting the use of proteasome inhibitors as radiosensitizers.

Increased levels of ROS have been documented in a variety of tumours [38] and further elevation of intracellular ROS in order to trigger cell death is a promising therapeutic strategy. It has been previously demonstrated that ROS are intermediates in the regulation of proteasome inhibitor-induced cell death and that cytotoxicity is partially blocked by antioxidants [11,19,26]. Furthermore, chemotherapy-induced peripheral neuropathy, a major dose-limiting effect of many commonly used cytotoxic agents, including platinum drugs, taxanes, and vinca alkaloids, as well as bortezomib, may be caused by ROS accumulation [27].

The extent to which ROS mediated the cytotoxicity induced by bortezomib and MG132 was evaluated by simultaneous treatment of cells with the antioxidants NAC or tiron. Proteasome activity, reportedly, is not affected by NAC [11]. However, we observed that NAC prevented bortezomib-induced toxicity, most likely by counteracting the toxicity of ROS. Although the generation of ROS has been reported in cancer cell lines exposed to MG132, this may be a cell-specific phenomenon and, moreover, was appreciable only in response to concentrations of MG132 (≥ 10 μM) more than ten times greater than the highest dose examined in the present study [39,40]. At concentrations of MG132 which were sufficient to inhibit proteasome activity and induce cytotoxicity, no protective effect of antioxidants nor generation of ROS was reported [31,35,40], consistent with the results of this study.

We also showed that bortezomib-induced but not MG132-induced toxicity was prevented by tiron, as has been previously demonstrated in melanoma cells [34]. Although it has been suggested that the superoxide scavenger tiron attenuated bortezomib-induced cell death through a ROS-dependent mechanism [41], polyhydroxyl compounds such as tiron also bind to boronic acid with high affinity [34], directly interfering with the proteasome-inhibitory function of bortezomib. This may account for tiron's abrogation of bortezomib-induced toxicity as well as the lack of effect on toxicity induced by non-boronated MG132. Direct binding of tiron to bortezomib may also explain the nullification of toxicity induced by high concentrations of bortezomib (> 10 nM) in SK-N-BE(2c) cells, wherein the toxicity was only partially reversed by NAC. Mechanistically, MG132-induced cytotoxicity may be caused by activation of the mitochondria-dependent caspase cascade, accumulation of pro-apoptotic proteins, suppression of NF-κB activation and cell cycle arrest in G2/M [33,36,42]. Therefore, although MG132 was toxic to cancer cells and enhanced radiation-induced cell kill in a manner similar to bortezomib, MG132 may have a reduced likelihood of ROS-related side effects.

## Conclusion

The experimental combination therapy studies reported here indicate the potential use of proteasome inhibition as a means of enhancing radiation-induced cancer cell kill, especially when given in combination with the radiopharmaceutical ^131^I-MIBG for the targeted radiotherapy of neuroblastoma. Bortezomib is routinely used in the treatment of haematological malignancies and has been reported to be well tolerated in children. Although it has been shown to be effective as a single agent in pre-clinical models of neuroblastoma, bortezomib is not only minimally effective in the treatment of various other solid tumours but also has been associated with toxic side effects when administered as a single agent. Therefore, consideration should be given to combination therapies including alternative proteasome inhibitors which are expected to have preferable clinical toxicity profiles.

## Competing interests

The authors declare that they have no competing interests.

## Authors’ contributions

CR designed and carried out the *in vitro* experiments, performed the analysis of the results and drafted the manuscript. MT carried out the xenograft experiments and performed the analysis of the results. MB and JWB made a substantial contribution to the conception, design and organisation of the conduct of the study. RJM contributed to the design, supervision, and preparation of the manuscript. All authors read and approved the final manuscript.
